# Development of a computational promoter with highly efficient expression in tumors

**DOI:** 10.1186/s12885-018-4421-7

**Published:** 2018-04-27

**Authors:** Shu-Yi Ho, Bo-Hau Chang, Chen-Han Chung, Yu-Ling Lin, Cheng-Hsun Chuang, Pei-Jung Hsieh, Wei-Chih Huang, Nu-Man Tsai, Sheng-Chieh Huang, Yen-Ku Liu, Yu-Chih Lo, Kuang-Wen Liao

**Affiliations:** 1 Department of Biological Science and Technology, National Chiao Tung University, Hsinchu, Taiwan, Republic of China; 2 Institute of Molecular Medicine and Bioengineering, National Chiao Tung University, Hsinchu, 30050 Taiwan, Republic of China; 3 Center for Bioinformatics Research, National Chiao Tung University, Hsinchu, Taiwan, Republic of China; 4 Institute of Bioinformatics and Systems Biology, National Chiao Tung University, Hsinchu, 300 Taiwan, Republic of China; 5 College of Biological Science and Technology, National Chiao Tung University, Hsinchu, Taiwan, Republic of China; 6 School of Medical and Laboratory Biotechnology, Chung Shan Medical University, Taichung, Taiwan, Republic of China; 7 Clinical Laboratory, Chung Shan Medical University Hospital, Taichung, Taiwan; 8 Department of Surgery, National Yang Ming University, Taipei, Taiwan, Republic of China; 9 Division of Colon and Rectal surgery, Department of surgery, Taipei Veteran General Hospital, Taipei, Taiwan, Republic of China; 10 Department of Biotechnology and Bioindustry Sciences, College of Bioscience and Biotechnology, National Cheng Kung University, Tainan, Taiwan, Republic of China; 11 Graduate Institute of Medicine, College of Medicine, Kaohsiung Medical University, Kaohsiung, Taiwan, Republic of China

**Keywords:** Gene therapy, Transcription factor, HIF-1α, NF-κB and CREB

## Abstract

**Background:**

Gene therapy is a potent method to increase the therapeutic efficacy against cancer. However, a gene that is specifically expressed in the tumor area has not been identified. In addition, nonspecific expression of therapeutic genes in normal tissues may cause side effects that can harm the patients’ health. Certain promoters have been reported to drive therapeutic gene expression specifically in cancer cells; however, low expression levels of the target gene are a problem for providing good therapeutic efficacy. Therefore, a specific and highly expressive promoter is needed for cancer gene therapy.

**Methods:**

Bioinformatics approaches were utilized to analyze transcription factors (TFs) from high-throughput data. Reverse transcription polymerase chain reaction, western blotting and cell transfection were applied for the measurement of mRNA, protein expression and activity. C57BL/6JNarl mice were injected with pD5-hrGFP to evaluate the expression of TFs.

**Results:**

We analyzed bioinformatics data and identified three TFs, nuclear factor kappa-light-chain-enhancer of activated B cells (NF-κB), cyclic AMP response element binding protein (CREB), and hypoxia-inducible factor-1α (HIF-1α), that are highly active in tumor cells. Here, we constructed a novel mini-promoter, D5, that is composed of the binding sites of the three TFs. The results show that the D5 promoter specifically drives therapeutic gene expression in tumor tissues and that the strength of the D5 promoter is directly proportional to tumor size.

**Conclusions:**

Our results show that bioinformatics may be a good tool for the selection of appropriate TFs and for the design of specific mini-promoters to improve cancer gene therapy.

**Electronic supplementary material:**

The online version of this article (10.1186/s12885-018-4421-7) contains supplementary material, which is available to authorized users.

## Background

Gene therapy has been widely regarded as a promising modality for the treatment of various cancers [1–3]. However, one of the problems with gene therapy is the low expression level of the transgene, leading to a negative impact on the efficacy of gene therapy. The secondary problem is that nonspecific expression between tumor tissues and normal tissues may cause side effects. Therefore, tumor-specific promoters have been considered to improve cancer gene therapy. Transcription factor response elements (TFREs) in the eukaryotic promoter control the strength and specificity of gene expression [4, 5]. In cancer cells, certain specific transcription factors (TFs) are overactive and substantially contribute to malignant progression [6]. Thus, tumor-specific TFREs were combined to form a tumor-specific mini-promoter that may enhance gene expression levels in tumor cells and reduce the side effects.

Recently, bioinformatics has been used to efficiently analyze abundant bio-information. In addition, free databases such as the Gene Expression Omnibus (GEO) and the Cancer Genome Atlas (TCGA) databases provide abundant clinical information and have been demonstrated to be useful in identifying new tumor marker genes or targeted treatment [7, 8]. On the other hand, there are many online databases and proteomic tools that can be used to analyze gene function and predict the pathways that they influence [9]. Using these methods, overexpression of E2F in gastric cancer [10], overexpression of SPP1 in metastatic prostate cancer [11], and overexpression of hub in colorectal cancer [12] were identified and confirmed. Therefore, bioinformatics may be useful to help identify tumor-specific TFREs that allow cancer cell-specific gene expression.

In this study, bioinformatics approaches were utilized to analyze the high-throughput data. Three TFs (NF-κB, CREB and HIF-1α) were identified and overexpressed in most types of cancer cells but not in normal cells. Therefore, the D5 mini-promoter was constructed by combining the three TFs, and the D5 promoter was shown to result in overexpression of the reporter gene in tumor tissues but not in normal tissues. Interestingly, the levels of reporter gene expression in tumor tissues were tumor size dependent. This study provides a convenient platform with which to identify suitable TFs for the construction of promoters, and the D5 tumor-specific promoter may improve the efficacy of cancer gene therapy in the future.

## Methods

### Bioinformatics analysis

The CEL files were composed of analyzed microarray data that were obtained using the Affymetrix GeneChip® Human Genome U133 Plus 2.0 Array and were retrieved from the *GEO* database [13]. The data were collected from patient samples of 8 cancers (breast cancer, colon cancer, lung cancer, melanoma, oral cancer, liver cancer, ovarian cancer and pancreatic cancer) and were normalized using the Robust Multiarray Analysis (RMA) algorithm [14, 15]. Data preprocessing and analysis were performed using the *‘affy’* and *‘stats’* packages in R software (http://www.r-project.org/) [16].

The 1624 TFs were defined using data from the TRANSFAC database (version 2012.4) [17], and the expression levels of these TFs were extracted from the expression data of 19,902 genes. Furthermore, TFs involved in cell growth or angiogenesis were selected by Gene Ontology (GO) [18]. One hundred and eleven TFs were shown to be associated with the functions of cell growth or angiogenesis (GO:0016049 for cell growth and GO:0001525 for angiogenesis). To illustrate the biological pathways in which the 111 TFs were involved, enrichment analysis was carried out via the Database for Annotation, Visualization and Integrated Discovery (DAVID) [19]. Afterward, the identified TFs with a log2 fold change ≥1 were chosen.

An online reference database (PubMed) was searched for the selected TFs. The following search keywords were used: “transcription factor gene and tumor and cell growth” or “transcription factor gene and tumor and angiogenesis.” The references were further identified to distinguish whether there was overlap in the two searches, and the number of references was calculated. Articles published before 2016 were included in the present study.

### Protein interaction (PPI) network, functional analysis of genes in the PPI network and protein expression

The PPI database GENEMANIA (http://genemania.org/) was used to obtain the interactions among the selected TFs, including NF-κB, CREB and HIF-1α. The proteins that interact with the selected TFs were predicted, and their gene names were obtained. These predicted genes were further verified by their related biological functions using UniProt (http://www.uniprot.org/). The protein expression levels were mined from “The Human Protein Atlas” (https://www.proteinatlas.org/) [20].

### Cell culture

The human cell lines MCF-7 (BCRC 60436), A-549 (BCRC 60074), AGS (BCRC 60102), HEK293 (BCRC 60019), and H184B5F5/M10 (BCRC 60197) were obtained from Bioresource Collection and Research Center (BCRC, Hsinchu, Taiwan). The human cell lines HT29 (ATCC® HTB38™) and HUVECs (ATCC® PCS-100-010 ™) were obtained from the American Type Culture Collection (ATCC, VA, USA). The human cell line PaTu8988T (ACC 162) was obtained from Deutsche Sammlung von Mikroorganismen und Zellkulturen (DSMZ, Braunschweig, German). The mouse cell lines B16F10 (BCRC 60031) and BALB/3 T3 (BCRC 60009) were obtained from the BCRC (Hsinchu, Taiwan). The human pancreatic duct epithelial cell line HPDE was kindly provided by Dr. Y.S. Shan. (National Cheng Kung University Medical College, Tainan, Taiwan), which are human papillomavirus-E6 and -E7 gene-immortalized pancreatic ductal epithelial cells [21]. HT29, MCF-7, A549, PaTu8988T, B16F10 and BALB/3 T3 cells were maintained in Dulbecco’s modified Eagle’s medium (DMEM; Invitrogen, CA, USA); HEK293 and H184B5F5/M10 cells were maintained in Minimum Essential Medium Eagle medium (MEM; Sigma-Aldrich, Shanghai, China); AGS and HPDE cells were maintained in RPMI medium 1640 (Invitrogen, CA, USA); and HUVECs were maintained in Medium 199 (Gibco, CA, USA) with 25 U/ml heparin and 30 μg/ml endothelial cell growth supplement (ECGS) in 5% CO_2_ at 37 °C. All media were supplemented with heat-inactivated 10% fetal bovine serum (Gibco, CA, USA) and 1% penicillin/streptomycin/amphotericin.

### Reverse transcription polymerase chain reaction (RT-PCR)

Total cellular RNA was extracted with TRIzol reagent (Life Technologies, Glasgow, UK) according to the manufacturer’s instructions. The total RNA was reverse transcribed into cDNA following the Superscript™-III kit (Invitrogen, CA, USA) instructions. The sequences of the primers used for PCR are shown in Additional file 1. The PCR products were analyzed on 2% agarose gels and photographed under a UV box after EtBr staining.

### Western blotting

Cells were collected and lysed in ice-cold RIPA lysis buffer. Sixty micrograms of protein was electrophoresed by SDS-PAGE using 10% polyacrylamide gels and transferred onto nitrocellulose membranes. The membranes were blocked with 5% skim milk in phosphate-buffered saline with 0.05% Tween 20 for 1 h. The membranes were probed using anti-NF-κB antibody (1:200; Santa Cruz, Shanghai, CN), anti-CREB antibody (1:500; GeneTex, CA, USA), anti-HIF-1α antibody (1:500; GeneTex, CA, USA) or anti-β-actin antibody (GeneTex, CA, USA) followed by anti-mouse or anti-rabbit secondary antibodies (1:10,000; GeneTex, CA, USA). The protein bands were developed using the ChemiLucent ECL Detection System (Millipore, MA) and were visualized using the Biospectrum AC Imaging System (UVP, CA, USA).

### Construction of the D5 mini-promoter

As shown in Fig. 4, the D5 mini-promoter was prepared as previously described [22]. The order of the 3 copies of each TF binding site in the sequence of the D5 promoter is NF-κB, CRB and HRE. The DNA fragments of the D5 promoter were assembled by PCR with the primers shown in Additional file 2, and the length was 103 bp. Furthermore, pD5-hrGFP was obtained by replacing the CMV promoter of pAAV-MCS-hrGFP with the D5 promoter. Briefly, the pAAV-MCS-hrGFP vector was double digested by MluI and ClaI (Fermentas, Burlington, Canada) to remove the sequence of the CMV promoter, and the DNA fragments of the D5 promoter were ligated into the digested vector.

### In vitro transcription factor activity assay

The assay plasmids (pHRE-hrGFP, pNF-κB-hrGFP, pCRE-hrGFP, pD5-hrGFP) and control plasmid (pARE-hrGFP; ARE is the binding site of a prokaryotic transcription factor ampR) were each co-transfected with a reporter plasmid (pAsRed2-N1, Clontech, CA, USA) into each type of cell. Cells (4 × 10^5^) were seeded into the wells of 6-well plates overnight and were transfected with different plasmids using Lipofectamine™ 2000 (Invitrogen, CA, USA) according to the manufacturer’s protocol. At 24 h after transfection, the cells were harvested and reseeded into the well of 24-well plates (2 × 10^5^ cells) followed by treatment with or without different activators (800 μM CoCl_2_, 10 ng/ml TNF-α or 400 nM PMA), incubation for 24 h at 37 °C and measurement of the fluorescence signal using a C6 flow cytometer (BD, CA, USA). The expression index of each transcription factor was calculated using the following formula:

1 $$ \mathrm{Expression}\ \mathrm{Index}=\frac{\mathrm{TFI}\ \mathrm{of}\ \mathrm{TFBS}\hbox{--} \mathrm{hrGFP}\div \mathrm{TFI}\ \mathrm{of}\ \mathrm{AsRed}2\ }{\mathrm{TFI}\ \mathrm{of}\ \mathrm{ARE}\hbox{--} \mathrm{hrGFP}\div \mathrm{TFI}\ \mathrm{of}\ \mathrm{AsRed}2} $$

where TFI is the total fluorescence intensity and TFBS is the transcription factor binding site.

### Construction of the pCMV-RBDV-IgG1 Fc, pCMV-IgG1-Fc, pD5-RBDV-IgG1 Fc and pD5-IgG1-Fc plasmids

Briefly, pCMV-RBDV-IgG1 Fc and pCMV-IgG1-Fc were constructed as previously described [23]. For pCMV-RBDV-IgG1 Fc, the RBDV (receptor binding domain of human vascular endothelial growth factor A (VEGF-A residues 1–109)) and the Fc region of human IgG1 were fused and cloned into the pAAV/MCS vector (Stratagene California, CA, USA). For pCMV-IgG1 Fc, the Fc region of human IgG1 was cloned into the pAAV/MCS vector (Stratagene California, CA, USA).

For pD5-RBDV-IgG1 Fc, the fragments of RBDV-IgG1 Fc were amplified from pAAV-RBDV-IgG1 Fc plasmids using the forward primer 5′-AAA GGT ACC TGA ACT TTC TGC TGT CTT GGG-3′ and reverse primer 5′-AAA AGA TCT TCA ATG GTG ATG GTG ATG ATG C-3′. The PCR products were generated with new restriction enzyme sites at the 5′-end (KpnI) and 3′-end (BglII) (underlined in the sequence of primer) and directly cloned into the pD5 vector to form pD5-RBDV-IgG1 Fc. For pD5- IgG1 Fc, the DNA fragments of IgG1 Fc were amplified from pAAV- IgG1 Fc plasmids using the forward primer 5′-AAA GGT ACC GTG GAA TTG CCC TTA TGT ACA G-3′ and reverse primer 5′-AAA AGA TCT TCA ATG GTG ATG GTG ATG ATG CG-3′, and the PCR products were cloned into the pD5 vector.

### Preparation of the LPPC and DNA/LPPC complexes

Briefly, the LPPC (liposome-PEG-PEI complex) particles were prepared as previously described [24]; their particle sizes and zeta-potential were subsequently evaluated. LPPC/DNA complexes were prepared by mixing 1 mg of LPPC with 100 μg of pAAV-MCS-hrGFP, pAAV-D5-hrGFP, pCMV-RBDV-IgG1 Fc, pCMV-IgG1-Fc, pD5-RBDV-IgG1 Fc or pD5-IgG1-Fc in 100 μl of H_2_O at 25 °C for 30 min.

### In vivo transfection

Female C57BL/6JNarl mice (8 weeks of age) were purchased from the National Laboratory Animal Center. All animal experiments were performed in accordance with and approved by the Institutional Animal Care and Use Committee at National Chiao Tung University (NCTU-IACUC-104034). For monitoring the expressions of specific transcriptional factors in different tumor sizes, B16F10 cells (1 × 10^6^) were implanted subcutaneously into C57BL/6JNarl mice. The mice were sacrificed when the tumors grew to 50 mm^3^, 100 mm^3^, 250 mm^3^, 500 mm^3^ or 1000 mm^3^. The tumors were harvested, fixed in 10% formalin, and embedded in paraffin. The sections (7 μm) of paraffin-embedded tumors were deparaffinized with xylene, rehydrated through alcohols, and stained with IHC to observe the expression level of HIF-1α, NF-κB and CREB.

For intra-tumor transfection, B16F10 cells (1 × 10^6^) were implanted subcutaneously into C57BL/6JNarl mice. The mice were injected with LPPC/pD5-hrGFP or LPPC/pCMV-hrGFP complexes when the tumors grew to 50 mm^3^, 100 mm^3^, 250 mm^3^, 500 mm^3^ or 1000 mm^3^. At 7 days after transfection, the mice were sacrificed by CO_2_ asphyxiation, and the tumors were obtained and embedded in OCT compound embedding medium (Sakura Finetek USA Inc., CA, USA) followed by storage at − 80 °C.

For intra-muscle experiments, the mice were intramuscularly injected with PBS or LPPC/pD5-hrGFP or LPPC/pCMV-hrGFP complexes into the tibialis anterior. At 7 days after transfection, the mice were sacrificed by CO_2_ asphyxiation, and the leg muscles were harvested and embedded in OCT compound embedding medium followed by storage at − 80 °C.

For normal organ experiments, the mice were injected intravenously with LPPC/pD5-hrGFP or LPPC/pCMV-hrGFP. At day 7, the mice were sacrificed by CO_2_asphyxiation, and the heart, liver, spleen, lung and kidney were collected and embedded in OCT compound embedding medium followed by storage at − 80 °C. Frozen tissue sections were examined and photographed by fluorescence microscopy (ZEISS AXioskop2).

Seven-micrometer-thick frozen sections were analyzed at ten random fields (200× magnification) per sample. The expression level of GFP was quantified and calculated as the GFP expression score. The GFP expression level was analyzed using ImageJ software. The GFP expression score was calculated as the fluorescence intensity × the fluorescent area. The intensities of the fluorescence signals were divided into 4 levels (0, 1, 2 or 3 levels). The fluorescent areas were defined as follows: 0–5% of the total area in the section = 0; 5–20% of the total area in the section = 1; 20–40% of the total area in the section = 2; 40–60% of the total area in the section = 3; 60–80% of the total area in the section = 4; and 80–100% of the total area in the section = 5.

### Immunohistochemical (IHC) staining

Paraffin-embedded sections (7 μm) of the different tumors or organs were obtained and processed for immunohistochemical staining. Briefly, after the dewaxing and rehydrating processes, the slides were treated with 3% hydrogen peroxide in 1× PBS for 10 min to block endogenous peroxidase activity. Next, the sections were washed three times with PBS-T (1× PBS containing 0.05% Tween-20) for 5 min per wash, and nonspecific reactions were blocked by 10% FBS in PBS for 10 min at room temperature. The sections were incubated with primary antibody (anti-NF-κB antibody, anti-CREB antibody and anti-HIF-1α antibody) overnight at 4 °C. Then, the sections were incubated with biotin-conjugated anti-mouse, anti-rabbit or anti-human IgG1 Fc antibody (1/1000 dilution) for 1 h at room temperature followed by incubation using the LSAB2 system (DAKO, CA, USA). After washing, 0.5 mg/ml diaminobenzidine (DAB) and 0.03% (*v*/v) H_2_O_2_ were added to develop the stain in PBS for 10 min. Finally, the sections were counterstained with hematoxylin, mounted and photographed by AXioskop2 microscopy (Zeiss, Jena, Germany). The protein expression scores were calculated as staining intensity × staining area. The intensities of expression were divided into 4 levels (0, 1, 2 or 3 levels). The expression areas were defined as follows: 0–5% of the total area in the section = 0; 5–20% of the total area in the section = 1; 20–40% of the total area in the section = 2; 40–60% of the total area in the section = 3; 60–80% of the total area in the section = 4; and 80–100% of the total area in the section = 5.

### In vivo safety assessment

The mice were injected intravenously with LPPC/pCMV-RBDV-IgG1 Fc, LPPC/pCMV-IgG1-Fc, LPPC/pD5-RBDV-IgG1 Fc or LPPC/pD5-IgG1-Fc complexes. At day 7, the mice were sacrificed by CO_2_ asphyxiation, and the heart, liver, spleen, lung and kidney were collected and fixed by paraformaldehyde. Finally, the tissue sections were stained with hematoxylin and eosin (H&E) or underwent IHC staining as previously described.

### In vivo tumor therapy

B16F10 cells (1 × 10^6^) were implanted subcutaneously in five female C57BL/6JNarl mice (8 weeks of age) per group. The mice were in situ injected with PBS or LPPC/pCMV-RDBV-IgG1 Fc, LPPC/pCMV-IgG1 Fc, LPPC/pD5-RDBV-IgG1 Fc or LPPC/pD5-IgG1 Fc complexes when the average tumor size reached 50 mm^3^. The tumor sizes of mice were measured every 2 days. Tumor sizes (mm^3^) were calculated as length × width × height. Mice were sacrificed at 21 days.

### Statistical analysis

The results were analyzed using the SAS statistical software package (SAS Institute Inc., Cary, USA). All of the results are expressed as the mean ± SD. A t-test was used to compare two independent trials. Differences of *p* < 0.05 were considered to be statistically significant.

## Results

### Design of the mini-promoter by bioinformatics

**Fig. 1:**
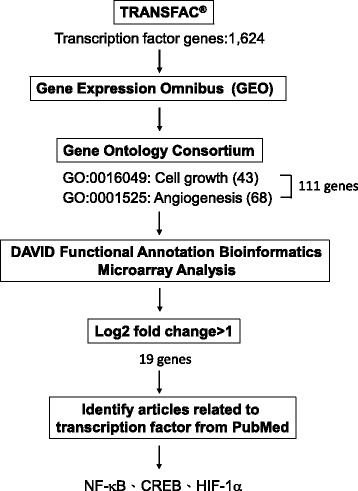
Flowchart of bioinformatics analysis for selecting the transcription factors

As previously discussed, we aimed to obtain a promoter that can overexpress transgenes in the growing tumor area. Therefore, we designed the sequence of this promoter by bioinformatics. Figure 1 displays the flow chart describing the study design. According to the TRANSFAC database, 1624 genes with TF activity were identified. Subsequently, the Gene Ontology Consortium was used to define whether the activity of these TFs was associated with cell growth. Since angiogenesis is also closely associated with tumor growth, the TFs with angiogenic activity were also selected. The number of genes involved in cell growth and angiogenesis was 43 and 68, respectively. A total of 8 genes overlapped in both groups, and they are displayed in Additional file 3. To further retrieve the biological pathways for the 111 TFs, enrichment analysis was carried out using the DAVID bioinformatics tools. As shown in Additional file 4, the enriched biological pathways were identified and divided based on carcinogenesis processes.

The expression data of 111 genes were mined from the GEO database; the expression levels of 19,902 genes in different tumor and normal samples were obtained. The analyzed microarray data include the following: breast cancer (GSE10780, GSE10810, GSE11001, GSE12276, GSE12790, GSE13787, GSE14020, GSE17907, GSE18728, GSE18864, GSE19697, GSE20086, GSE20713, GSE21422, GSE22840, GSE23640, GSE29431, GSE31138, GSE31448, GSE3744, GSE42568, GSE43365, GSE47109, GSE5460, GSE5764, GSE6532, GSE7515, GSE7904 and GSE8977), colon cancer (GSE10961, GSE13067, GSE13294, GSE13471, GSE15960, GSE17538, GSE18105, GSE18462, GSE20916, GSE22598, GSE23878, GSE33371, GSE37364, GSE39582, GSE4107, GSE41328, GSE4183 and GSE9348), lung cancer (GSE10245, GSE10445, GSE10799, GSE12345, GSE12667, GSE18842, GSE19188, GSE27262, GSE30219, GSE33356 and GSE43346), melanoma (GSE15605, GSE31879 and GSE7553), oral cancer (GSE29330, GSE30784, GSE38517, GSE42743 and GSE51010), liver cancer (GSE17548, GSE19665, GSE29722, GSE33006, GSE41804, GSE6222, GSE6465, GSE6764 and GSE9829), ovarian cancer (GSE10971, GSE12172, GSE14001, GSE14407, GSE15578, GSE18520, GSE19352, GSE27651, GSE29450, GSE36668, GSE38666 and GSE9899) and pancreatic cancer (GSE15471, GSE16515, GSE18670, GSE19650, GSE22780 and GSE32688). After calculation, genes were selected if their fold change in the tumor vs. normal sample was greater than 2-fold (log2 > 1). Following such criteria, 19 genes were selected, and their importancewas evaluated by searching key words in the PubMed database to calculate the number of studies in which the genes were associated with tumor growth or angiogenesis (Table 1). The results showed that 9 TFs were well studied and published in more than 100 articles. The top 3 selected TFs were HIF-1α, CREB and NF-κB.

**Table 1:** PubMed articles related to the 19 genes

Gene name	Function		Cell growth plus angiogenesis	Cell growth or angiogenesis
	Cell growth	angiogenesis		
*ELK3*	11	5	2	14
*HEY1*	79	21	17	83
*HIF1A*	2852	1506	1113	3245
*HMGB1*	313	46	30	329
*HOXB3*	14	3	3	14
*ID1*	294	85	55	324
*KLF5*	86	11	3	94
*UBP1*	1	0	0	1
*VEZF1*	2	0	0	2
*ABL1*	117	14	8	123
*ADNP*	5	0	0	5
*CREB1*	751	64	43	772
*ENO1*	41	7	6	42
*IGFBP1*	327	13	9	331
*SMARCA4*	96	4	2	98
*SOX9*	182	11	6	187
*TAF9*	4	0	0	4
*WT1*	581	42	27	596
*NFKB1*	7373	967	678	7662

**Fig. 2:**
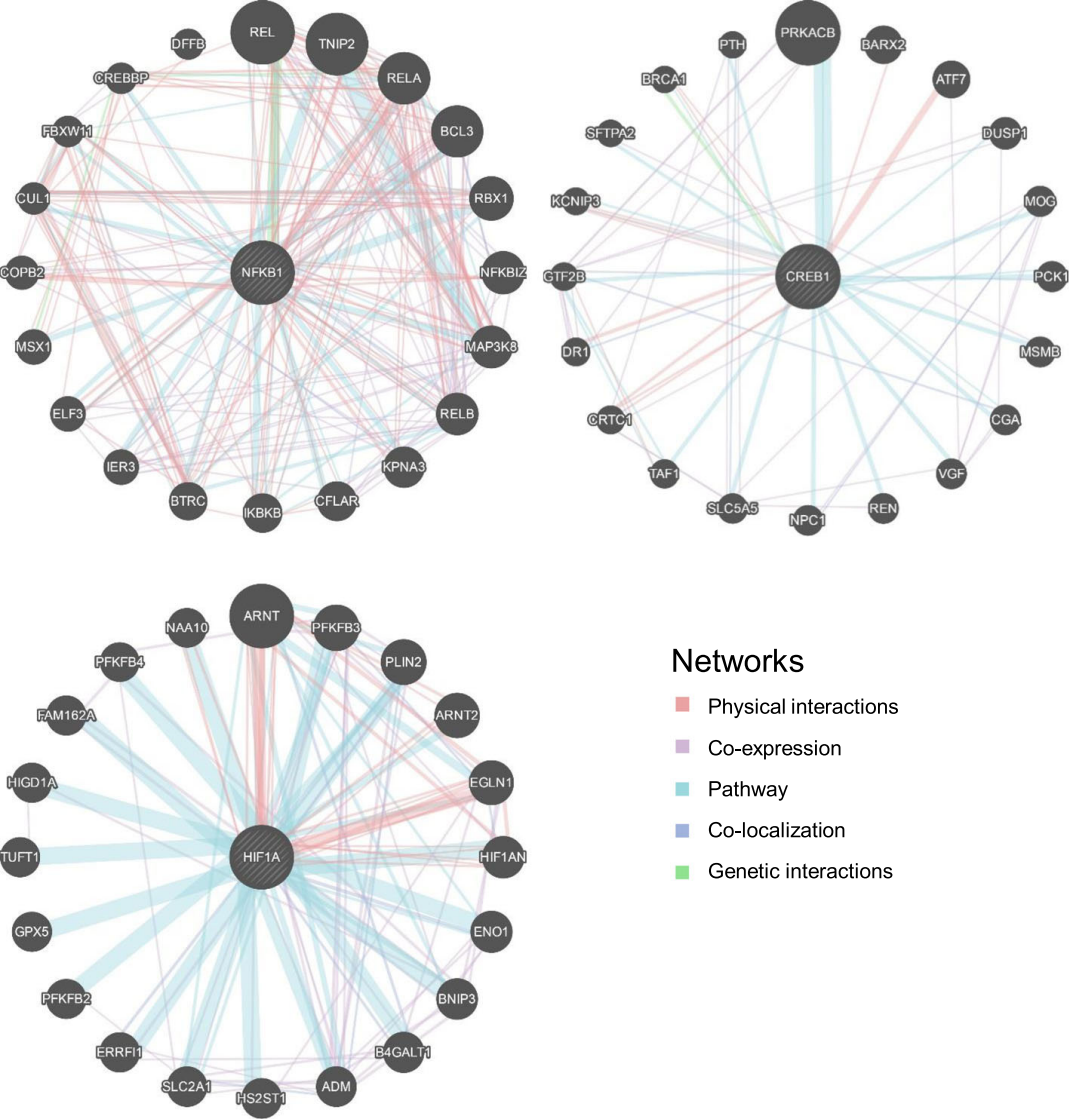
Analysis of NF-κB, CREB and HIF-1α. Prediction of the protein-protein interactions of NF-κB, CREB and HIF-1α. The predictions were analyzed by GENEMANIA. The pink line indicates a physical interaction, which is the highest interaction in the network. The purple, light blue, dark blue and green lines indicate that the protein interacts with the TF, with different effects, co-expression, involved pathways, co-localization and genetic interactions, respectively

Subsequently, the interactions involving these TFs were further analyzed using GENEMANIA. Figure 2 shows the proteins that could interact with NF-κB, CREB or HIF-1α. Using UniProt analysis, the functions of the gene products that interact with NF-κB suggest that they are involved in cell growth, cell death and inflammation, the functions of the gene products that interact with CREB suggest that they are involved in cell growth, apoptosis, angiogenesis and cell metabolism, and the functions of the gene products that interact with HIF-1α suggest that they are involved in cell growth, apoptosis, angiogenesis and cell metabolism (Additional file 5). In addition, the genes that cooperate with NF-κB, CREB and HIF-1α were analyzed with the GENEMANIA database. The results revealed that NF-κB has direct interactions with HIF-1α through physical interactions and co-expression and has direct interactions with CREB through co-expression. In addition, CREB has indirect interactions with NF-κB and HIF-1α via p300-CBP coactivator (CREBBP and EP300), which increases the expression level of their target genes (Additional file 6).

**Fig. 3:**
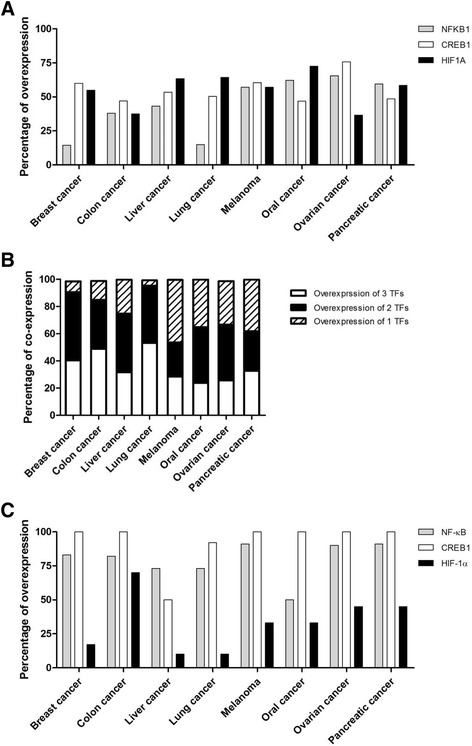
Overexpression of NF-κB, CREB and HIF-1α. **a** Percentages of overexpression for the NFKB1, CREB1 or HIF1A genes in eight cancers. The data were mined from the GEO database and were calculated to display the overexpression percentages of different TFs in different cancers. **b** Percentages of co-expression for the *NFKB1*, *CREB1* or *HIF1A* genes. Percentages of patients with co-expression or non-co-expression are displayed for the eight types of cancers. **c** Percentages of overexpression for the NF-κB, CREB or HIF-1α proteins in eight cancers. The data were mined from “The Human Protein Atlas” database and were calculated to display the percentages of overexpression among different TFs in different cancers

**Fig. 4:**
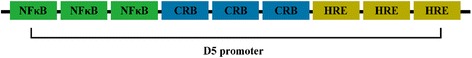
Schematic diagram of the D5 mini-promoter. The D5 mini-promoter contained three copies of the NF-κB, CREB and HIF-1α binding sites (NF-κB, CRB and HRE)

Furthermore, we calculated the percentage of overexpression for these TFs in the samples of patients with different tumors for which the data were mined from the GEO database. The results reveal that all eight tumor types overexpressed at least one TF in over 50% of the patients (Fig. 3a). Figure 3b further shows that the frequencies at which tumors overexpress two or more of the three TFs were higher than the frequencies at which tumors overexpress only one of the three TFs. Moreover, The Human Protein Atlas database was used to analyze the protein expression levels of the three TFs. Figure 3c shows that the NF-κB and CREB proteins were overexpressed in over 50% of patients with the eight tumor types; HIF-1α was overexpressed in over 50% of patients with one tumor type and was significantly overexpressed in patients with other tumor types. Therefore, we designed a mini-promoter using the binding sequences of HIF-1α, CREB and NF-κB. The sequence of the D5 promoter comprises three copies of each of these TFs, and the construct is shown in Fig. 4.

### Differences in the activities of TFs between the tumor cells and normal cells in vitro

**Fig. 5:**
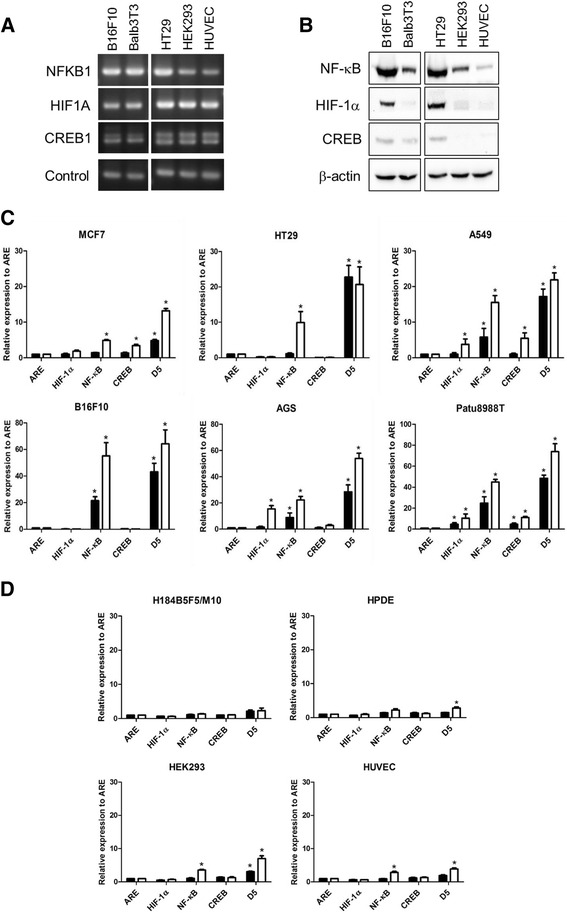
Expression level of transcription factors in different cells. **a** The mRNA levels of NF-κB, CREB and HIF-1α in B16F10, Balb3T3, HT29, HEK293 and HUVECs were determined by RT-PCR. The loading control for B16F10 and Balb3T3 cells was mouse β-actin, and the loading control for HT29, HEK293 and HUVECs was human GAPDH. **b** Protein expression levels of five cell lines were verified by western blotting. **c** and **d** In vitro verification of the D5 promoter activity in tumor and normal cell lines. pARE-hrGFP, pHIF-1α-hrGFP, pNFκB-hrGFP, pCRE-hrGFP and pD5-hrGFP were separately transfected into different cells. Twenty-four hours after transfection, the cell lines were treated with control medium (close column) or activators (open column). After the treatments, the green fluorescence levels were determined by a flow cytometer. The data were analyzed from three independent experiments, and statistically significant differences were determined by t-test (* *p* < 0.05)

We proposed that the D5 mini-promoter would drive transgene overexpression in tumors. Therefore, we verified the expression profile of the D5 promoter. The expression levels of the TFs NF-κB, CREB and HIF-1α were first examined in different cells. The results showed that except for tumor cell line HT29, tumor cells exhibited higher transcription levels of NF-κB than the normal cells HEK293 and HUVECs in the culture system. Similar gene expression levels of HIF-1α and CREB were observed between the tumor cell lines (HT29 and B16F10 cells) and normal cell lines (HUVECs and HEK293 and Balb3T3 cells), but HT29 cells had higher expression levels of NF-κB than HEK293 cells and HUVECs (Fig. 5a). However, the tumor cell lines HT29 and B16F10 exhibited higher protein expression levels of NF-κB, HIF-1α or CREB than normal cell lines, including HUVECs and HEK293 and Balb3T3 cells (Fig. 5b). Based on the expression level of the reporter gene EGFP driven by different mini-promoters, the activities of TFs were measured in different tumor and normal cells. Figure 5c shows that in the presence or absence of activator treatments, different mini-promoters exhibit different activity levels in different tumor cells (6 tumor cell lines were examined). With activator treatment, NF-κB binding element promoter activity in 100% of cell lines (6/6), HIF-1α binding element promoter activity in 50% of cell lines (3/6) and CREB binding element promoter activity in 50% of cell lines (3/6) were significantly higher than the activity level of the control ARE (binding site of a prokaryotic transcription factor ampR) promoter, which is a prokaryotic promoter with nonspecific expression in eukaryotic cells. Under such conditions, the D5 mini-promoter exhibited high activity levels and induced high gene expression levels in 6 tumor cell lines (Fig. 5c) but not in 4 normal cell lines (Fig. 5d). In addition, the CMV promoter was compared with the D5 mini-promoter, and Additional file 7 shows that the CMV promoter induced higher gene expression in the HEK293 cell line than the D5 mini-promoter; meanwhile, the CMV promoter did not induce significantly different gene expression levels in the B16F10 cell line compared with the D5 mini-promoter but did induce less gene expression in the HT29 cell line than the D5 mini-promoter. After the addition of different inhibitors, the activity of the D5 mini-promoter was significantly reduced (Additional file 8), revealing that the activity of each transcription factor in the D5 mini-promoter affects the strength of the D5 mini-promoter.

### In vivo activities of the D5 mini-promoter in tumors of different sizes or normal tissues

**Fig. 6:**
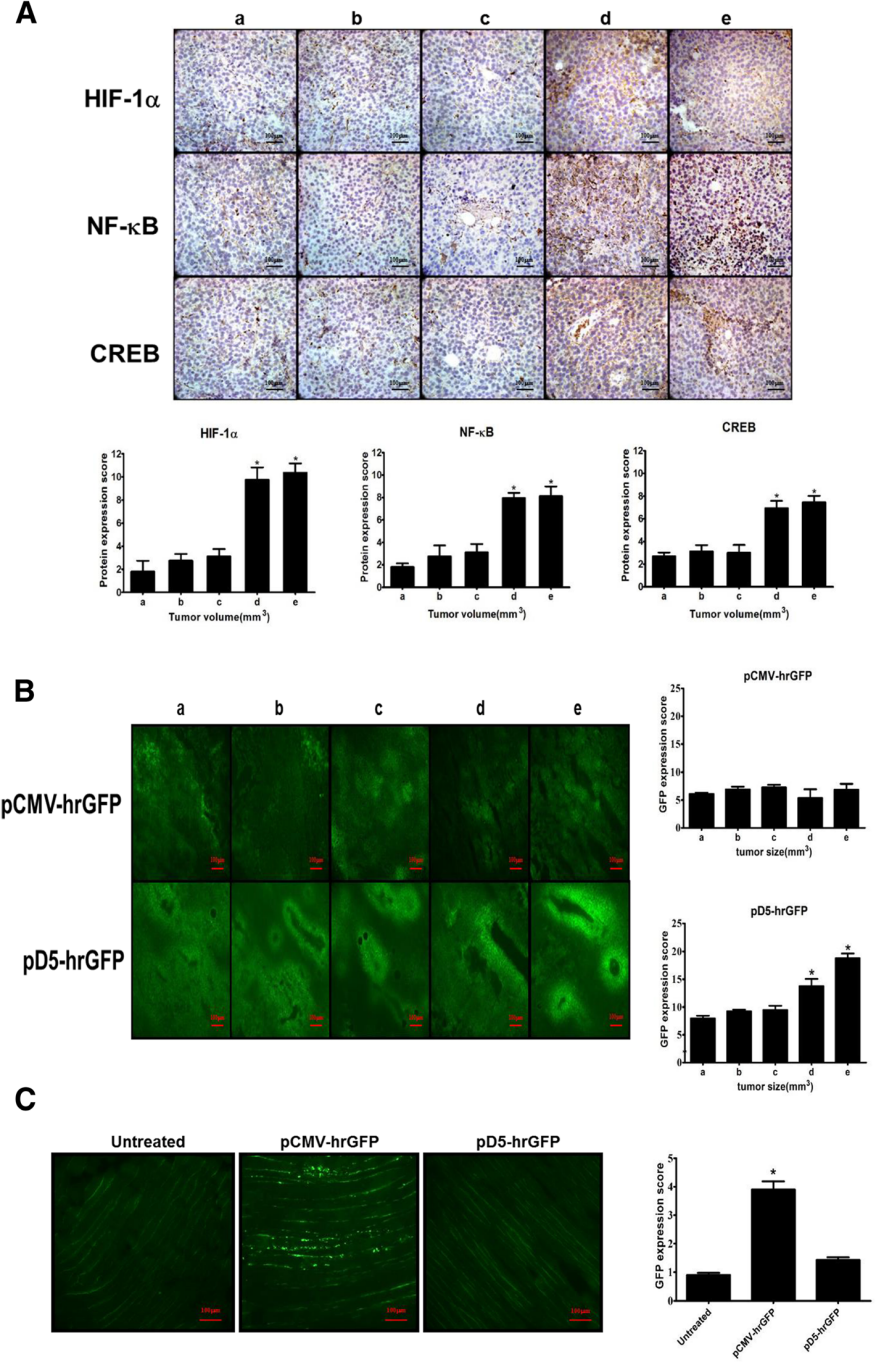
Expression of different transcription factors in tumors of different sizes. **a** Immunohistochemical (IHC) staining of HIF-1α, NF-κB or CREB in different-sized B16F10 tumors. The tissue sections of tumors were probed with primary and HRP-conjugated antibodies, developed and photographed under a microscope (Scale bar: 100 μm, 200×). According to the levels of brown-colored staining in the tumor tissue sections, the expression levels of the TFs were quantified and calculated as protein expression scores. The protein expression scores for the groups with different tumor sizes (a: 50 mm^3^, b: 100 mm^3^, c: 250 mm^3^, d: 500 mm^3^ and e: 1000 mm^3^) were calculated and are displayed for HIF-1α, NF-κB or CREB. Statistically significant differences were determined by t-test, and the *p* values were presented as the tested group compared with the control group (*: *p* < 0.05). **b** After intra-tumor transfections with pCMV-hrGFP or pD5-hrGFP in different-sized B16F10 tumors for 7 days, the green fluorescent protein levels in the B16F10 tumors were observed under a fluorescence microscope (Scale bar: 100 μm, 200×) and photographed. The GFP expression scores were estimated and calculated according to the different tumor sizes (a: 50 mm^3^, b: 100 mm^3^, c: 250 mm^3^, d: 500 mm^3^ and e: 1000 mm^3^). Statistical analysis of the average score of green fluorescent protein expression in B16F10 tumors was performed by t-test (*p < 0.05). **c** After intra-muscle transfection with pCMV-hrGFP or pD5-hrGFP for 7 days, the green fluorescent protein levels in muscles were observed under a fluorescence microscope (Scale bar: 100 μm, 200×) and photographed. The GFP expression scores were estimated and calculated. Statistical analysis of the average green fluorescent protein expression score in the muscles was performed by t-test (*p < 0.05)

**Fig. 7:**
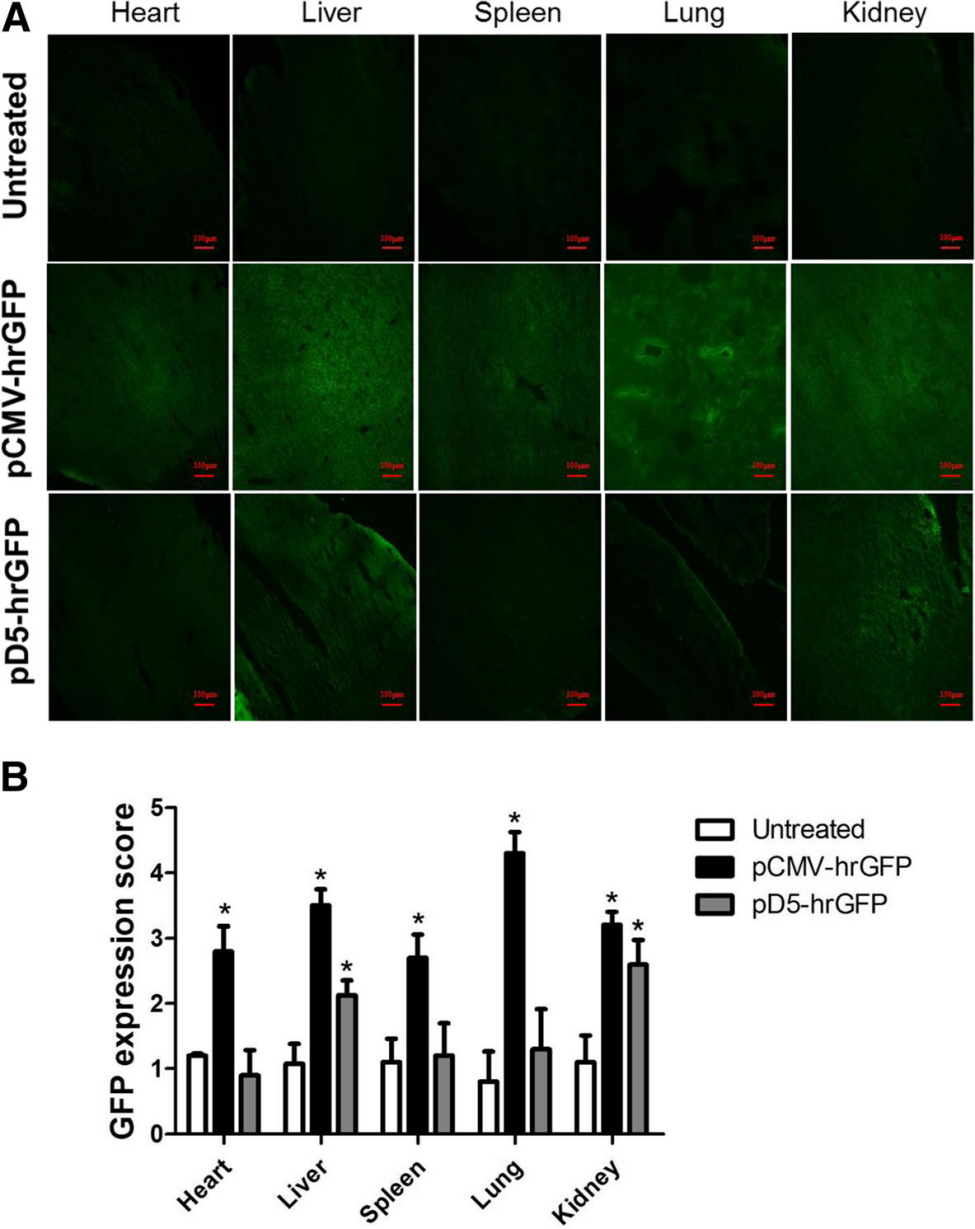
D5 promoter-driven expression of the hr-GFP reporter gene in normal organs. **a** Seven days after the transfection of pCMV-hrGFP or pD5-hrGFP via the lateral tail vein, the expression levels of green fluorescent proteins (GFP) in normal organs (heart, liver, spleen, lung, and kidney) were observed under a fluorescence microscope and photographed (Scale bar: 100 μm, 200×). **b** The GFP expression scores were calculated and compared. Statistical analysis of the average score of GFP in different normal organs was performed by t-test (*p < 0.05)

The levels of different TFs (HIF-1α, NF-κB and CREB) were examined in tumors of different sizes. The results showed that there were no differences in the expression levels of all TFs in tumors whose sizes were smaller than or equal to 250 mm^3^_,_ but all TFs showed increased expression in tumors whose sizes were greater than or equal to 500 mm^3^ (Fig. 6a). Subsequently, D5 promoter activity was examined in tumors of different sizes. The pathological results indicated that intratumoral injections of pCMV-hrGFP generally resulted in low expression levels of hrGFP protein, but injections of pD5-hrGFP resulted in strong expression of hrGFP protein, especially near angiogenic vessels. In addition, detection of the fluorescence signal from hrGFP revealed that the D5 promoter not only induced higher reporter gene expression levels than the CMV promoter but also induced gene expression in a tumor size-dependent manner (Fig. 6b). In contrast, intramuscular injections of pCMV-hrGFP induced stronger expression of hrGFP proteins in muscular cells than in untreated muscular cells, but pD5-hrGFP did not induce a significantly different change in hrGFP expression compared with that in untreated cells (Fig. 6c). Systemic transfection with pCMV-hrGFP or pD5-hrGFP was performed to further monitor the level of reporter gene expression in different organs. The results also showed that pCMV-hrGFP induced high hrGFP expression levels in liver and lung tissues and moderate hrGFP expression levels in the kidney, heart and spleen tissues. Unlike pCMV-hrGFP, pD5-hrGFP induced mild hrGFP expression in only liver and kidney tissues (Fig. 7a and b). These results indicate that the CMV promoter can drive gene expression in normal cells as well as tumor cells, but the D5 promoter could lead to divergent gene expression levels in tumor cells and normal cells.

### Tumor-inhibitory effect of D5 mini-promoter-driven expression of the therapeutic gene RBDV IgG1 Fc

**Fig. 8:**
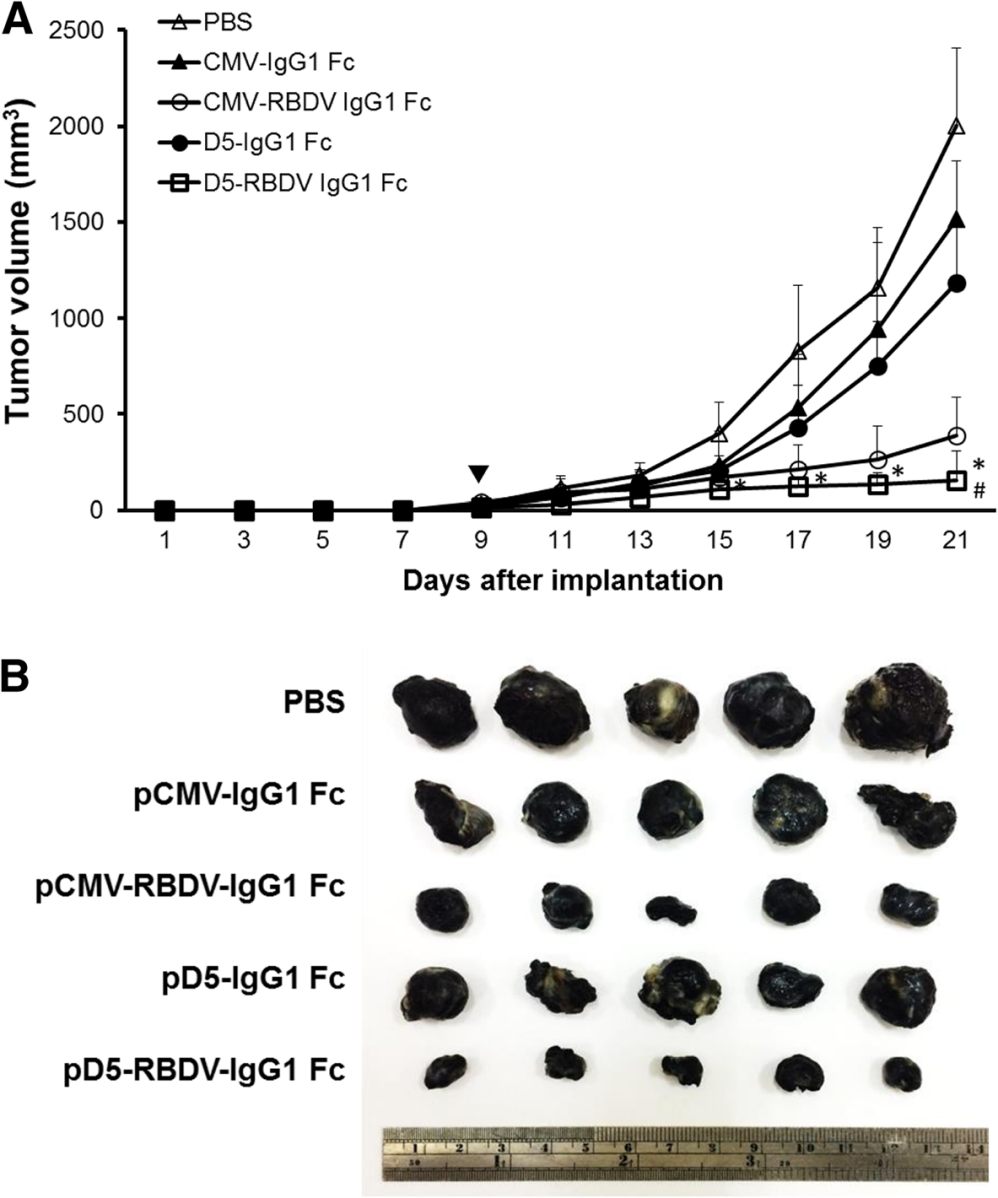
Inhibitory effect of D5 mini-promoter-driven expression of the therapeutic gene RBDV IgG1 Fc on tumor growth. **a** C57BL/6 mice (5 animals per group) were subcutaneously implanted with 1 × 10^6^ B16F10 tumor cells/mouse. When the average tumor sizes reached 50 mm^3^, the mice were in situ injected with PBS, or LPPC/pCMV-RDBV-IgG1 Fc, LPPC/ pCMV-IgG1 Fc, pD5-RDBV-IgG1 Fc or LPPC/pD5-IgG1 Fc complexes. Tumor growth was monitored every 2 days, and tumor sizes were calculated as described in the Methods section. For the pD5-RBDV-IgG1 Fc group, * indicates *p* < 0.05 compared with the PBS group and # indicates *p* < 0.05 compared with the pCMV-RBDV-IgG1 Fc group. **b** B16F10 tumors were collected and photographed after the mice were sacrificed at 21 days

To further evaluate the feasibility of using the D5 promoter for in vivo tumor therapy, we constructed a therapeutic gene, RBDV-IgG1 Fc, that codes a fusion protein for amino acid residues 8–109 of VEGF-A and the Fc region of human IgG1. RBDV-IgG1 Fc can inhibit tumor angiogenesis by binding to VEGF receptor 1 or 2. In this study, we established subcutaneous tumors in C57BL/6 mice using B16F10 cells and treated the tumors with D5 or CMV promoter-driven RBDV expression via in situ injections of D5-RBDV-IgG1 Fc or CMV-RBDV-IgG1 Fc, which caused significant tumor growth inhibitory effects compared to injections of D5-IgG1 Fc or CMV-IgG1 Fc after 17 days. In addition, D5-RBDV-IgG1 Fc exhibited better therapeutic efficacy than CMV-IgG1 Fc at 21 days after tumor inoculation (Fig. 8a). Moreover, D5-RBDV-IgG1 Fc treatments resulted in smaller tumor sizes than all other treatments (Fig. 8b).

In addition, the side effects of utilizing the D5 promoter were examined using in vivo biodistribution studies. The results showed no acute tissue damages in the organs (Additional file 9A). In addition, the expression levels were further monitored in different organs. According to the results of the GFP experiments, pCMV-driven genes were highly expressed in the liver and moderately expressed in the heart, lung, kidney and intestine tissues. Unlike pCMV-driven genes, pD5-RBDV-IgG1 Fc or IgG1 Fc was only mildly expressed in liver and kidney tissues (Additional file 9B and C). These results indicate that the D5-driven therapeutic genes could be specifically expressed in the tumor area and not in normal organs.

## Discussion

This study provided a convenient and efficient method to design a mini-promoter that could drive transgene overexpression in the tumor area but not in normal tissues. To obtain a promoter that can overexpress a transgene in the growing tumor area, we proposed a model (Fig. 1) using bioinformatics tools. Using this model, a D5 mini-promoter was designed to enable transgene overexpression in tumors in a size-dependent manner (Fig. 6b) while driving low transgene expression in normal organs (Figs. 6c and 7). Three TFs, HIF-1α, NF-κB and CREB, were proposed as elements of the D5 mini-promoter. As predicted, the D5 mini-promoter displayed specific expression in the tumor areas but not in normal tissues, as indicated by the calculations of fold change in the tumor vs. normal samples in the bioinformatics prediction step. In addition, the information from Fig. 3a and b revealed that most tumors overexpressed more than one TF among NF-κB, CREB and HIF-1α, possibly making the D5 mini-promoter stronger than other mini-promoters composed of a single TFRE (Fig. 5c). In addition, the TFs may cooperatively enhance gene expression [25]. Therefore, the interactions of the selected TFs were analyzed, and the predictions showed that there were direct and indirect interactions among NF-κB, CREB and HIF-1α (Additional file 6) that may suggest strong activity from the D5 mini-promoter.

As predicted, the results showed that the three predicted TFs exhibited high expression and activity in tumor cells in vitro (Fig. 5b and c) and in vivo (Fig. 6a). The pathological results also revealed that NF-κB, CREB and HIF-1α were overexpressed in tumors in a size-dependent manner (Fig. 6a), which may be the main reason for the tumor size-dependent activity of the D5 mini-promoter. Solid tumors need blood vessels to support their growth; an approximate 0.2-mm distance to the blood vessel is the limitation for efficient oxygen diffusion to maintain cell survival [26, 27]. Consequently, the growing solid tumors are exposed to hypoxic conditions that will facilitate a gene expression profile supporting angiogenesis and oxygen delivery through overexpression of HIF-α and NF-κB [28]. The literature also indicates that prolonged hypoxia will activate CREB and NF-κB and that these proteins will cooperate to induce the expression of MMP1 [29]. Thus, tumors with larger sizes would experience more hypoxic stress and exhibit higher expression and promotor activity levels of NF-κB, CREB and HIF-1α than small-sized tumors, resulting in the size-dependent expression of the D5 mini-promoter.

Moreover, our results showed that it was difficult to observe D5 promoter-derived gene expression in the tissues of normal organs, whereas CMV promoter-derived expression was notable (Figs. 6c and 7). The D5 promoter is composed of NF-κB, CREB and HIF-1α TFREs, which are regulated in normal cells. HIF-1α is rapidly degraded through the pVHL pathway under normoxic conditions [28], the activity of NF-κB is inhibited by I-κB until IKKs are activated [30], and phosphorylation at ser133 is necessary for CREB activation [31], all of which explain the lower activity of the D5 mini-promoter in normal tissues. In contrast, the CMV promoter is composed of multiple TFREs, including NF-κB, CREB, YY1, retinoic acid receptor and SP-1, and is repressed by p53 and activated by JNK [32]. Thus, it may be that many TFs can bind to the CMV promoter and initiate transcription to result in constitutive activation in both primary and transformed cells. Therefore, the D5 promoter is better than the CMV promoter for tumor-specific gene expression.

In general, the specific signaling pathways and TFs in tumors should be studied thoroughly, as the knowledge can be used to design a tumor-specific promoter. For example, telomerase (TERT) activation is a fundamental step in tumorigenesis, and many mutations in the TERT promoter are found in over 50 cancer types; additionally, TERT mutations are the most common mutations in many cancers [33]. Therefore, the hTERT promoter was used to drive the expression of IL-18 and HSV-TK in murine colorectal cancer cells as a novel cancer vaccine [34]. In addition, many tumor-specific promoters or TFRE have been identified, and they have been shown to exhibit cancer-specific expression, including the 5′-UTR of basic fibroblast growth factor-2 or the enhancer element targeted by beta-catenin [35]. In addition, the ETS-related gene (ERG), a member of the E-26 transformation-specific (ETS) family of TFs, is a key factor in prostate cancer [36], and the SP1 factor is a good target for anti-cancer proliferation [37]. Therefore, bioinformatics information can be easily integrated to design the required promoter. In addition, we showed that bioinformatics can be a convenient and effective tool for rapidly designing a promoter sequence for specific expression based on the vast knowledge in the literature.

## Conclusions

In summary, this study provides a convenient platform with which to identify suitable TFs for the construction of promoters, and the D5 promoter has the potential to reach optimal therapeutic effects with limited side effects for application in cancer gene therapy.

## Additional files

Additional file 1: Gene-specific primers for RT-PCR. (PDF 102 kb)

Additional file 2: D5 mini-promoter primers. (PDF 101 kb)

Additional file 3: The genes of regulate angiogenesis and cell growth. (PDF 102 kb)

Additional file 4: Pathway analysis of 111 TFs. (XLSX 12 kb)

Additional file 5: The genes function of protein-protein interaction with NF-κB, CREB, HIF-1α. (XLSX 14 kb)

Additional file 6: Cooperativity analysis of NF-κB, CREB and HIF-1α. The protein-protein interaction of (A) NF-κB with CREB, (B) NF-κB with HIF-1α, and (C) CREB with HIF-1α were analyzed by GENEMANIA. The pink line indicates physical interaction, which is the highest interaction in the network. The purple, light blue, dark blue and green lines indicate that the protein has an interaction with the TF with different effects, co-expression, the involved pathway, co-localization and genetic interaction, respectively. (PDF 212 kb)

Additional file 7: The expressive capabilities of D5 mini-promoter and CMV promoter in HEK293, B16F10 and HT29 cells. pD5-hrGFP or pCMV-hrGFP were transfected into (A) HEK293, (B) B16F10 or (C) HT29 cells 24 h, and the GFP expression intensities were detected by flow cytometer. The data were analyzed from three independent experiments, and the significant differences were calculated by t-test (* *p* < 0.05). (PDF 196 kb)

Additional file 8: The effects of inhibitors for transcription factors on the hrGFP expression levels in PaTu8988T cells. pARE-hrGFP, pHIF-1α-hrGFP, pNFκB-hrGFP, pCRE-hrGFP and pD5-hrGFP were transfected into PaTu8988T cells, respectively. The pD5-hrGFP-transfected cells were treated respectively with different inhibitors, 30 μM Bay11-7082 (NFκB inhibitor), 1 μg/ml DMGF (CREB inhibitor) and 1 atm oxygen. Twenty-four hours after transfection, the green fluorescent levels of hrGFP were determined by flow cytometer. The data were calculated and analyzed from three independent experiments, and the significant differences were calculated by t-test for the pD5-hrGFP transfected cells v.s. the pD5-hrGFP transfected cells treated with inhibitor (*p < 0.05). (PDF 233 kb)

Additional file 9: Histopathologic analysis of mice treated with LPPC/DNA complexes. (A) H&E staining of normal organs including the heart, liver, spleen, lung, kidney, intestine and stomach at day 7 after an intravenous injection of PBS, LPPC/pCMV-RDBV-IgG1 Fc, LPPC/ pCMV-IgG1 Fc, pD5-RDBV-IgG1 Fc or LPPC/pD5-IgG1 Fc complexes. (Scale bar: 50 μm, 400×) (B) IHC staining of normal organs using anti-Human IgG1 Fc antibody. (Scale bar: 50 μm, 400×) (C) Statistical analysis of the average score of human IgG1 Fc staining normal organs. Significance differences were evaluated by t-test, and the *p* values were represented as the tested group compared with the control group (*: *p* < 0.05). (PDF 1022 kb)

## Abbreviations

CREB: Cyclic AMP response element binding protein
ERG: ETS-related gene
ETS: E-26 transformation-specific
GEO: Gene Expression Omnibus
GO: Gene Ontology
H&E: Hematoxylin and eosin
HIF-1α: Hypoxia-inducible factor-1
IHC: Immunohistochemical
LPPC: Liposome-PEG-PEI complex
NF-κB: Nuclear factor kappa-light-chain-enhancer of activated B cells
PPI: Protein-protein interaction
RMA: Robust Multiarray Analysis
TCGA: The Cancer Genome Atlas
TFREs: Transcription factor response elements
TFs: Transcription factors
