# Supplementary material for: Development of a computational promoter with highly efficient expression in tumors
Source: BMC Cancer. 2018 Apr 27;18:480. doi: 10.1186/s12885-018-4421-7 (PMC5924487; doi:10.1186/s12885-018-4421-7)
Supplement: Supplementary file 1 — Gene-specific primers for RT-PCR. (PDF 102 kb) [file 12885_2018_4421_MOESM1_ESM.pdf]

Additional file 1. Gene-specific primers for RT-PCR

Gene	species	Orientation	Sequence
NFKB1	Human	Forward	5'-ATGGCAGAAGATGATCCATATTTG-3'
		Reverse	5'-TTTTCCCGATCTCCCAGCTGC-3'
HIF1A	Human	Forward	5'-ACGACAAGAAAAAGATAAGTTCTGA-3'
		Reverse	5'-TGCAGTGCAATACCTTCCATGTT-3'
CREB1	Human	Forward	5'-TGACCATGGAATCTGGAGCCG-3'
		Reverse	5'-TGGGTAATGGCAATATACTGTCC-3'
GAPDH	Human	Forward	5'-CCAGCCGAGCCACATCGCTC-3'
		Reverse	5'-ATGAGCCCCAGCCTTCTCCAT-3'
NFKB1	Mouse	Forward	5'-ATGGACGATCTGTTTCCCCT-3'
		Reverse	5'-ACCTCAATGTCTTCTTTCTGC-3'
HIF1A	Mouse	Forward	5'-AGAACGAGAAGAAAAAGATGAG-3'
		Reverse	5'-CATCAACTCAGTAATTCTTTCAT-3'
CREB1	Mouse	Forward	5'-ATGACCATGGAATCTGGAGC-3'
		Reverse	5'-ATCGCCTGAGGCAGCTTGA-3'
ACTB	Mouse	Forward	5'-TTGGGTATGGAATCCTGTGG3-3'
		Reverse	5'-TCGTACTCCTGCTTGCTGAT-3'
