# Supplementary material for: Development of a computational promoter with highly efficient expression in tumors
Source: BMC Cancer. 2018 Apr 27;18:480. doi: 10.1186/s12885-018-4421-7 (PMC5924487; doi:10.1186/s12885-018-4421-7)
Supplement: Supplementary file 2 — D5 mini-promoter primers. (PDF 101 kb) [file 12885_2018_4421_MOESM2_ESM.pdf]

Additional file 2. D5 mini-promoter primers

Name	Sequence 5' to 3'
5'-D51	CGCGTGGGACTTTCCGCTGGGGACTTTCCGCTGGGGACTTTCCGCTG**TGACGTCA**GAGAG
3'-D52	**TCA**GCTCTC**TGACGTCA**CAGCGGAAAGTCCCCAGCGGAAAGTCCCCAGCGGAAAGTCCCA
5'-D53	C**TGACGTCA**GAGAGC**TGACGTCA**GAGAGCTACGTGTGTGTACGTGTGTGTACGTGAT
3'-D54	CGATCACGTACACACACGTACACACACGTAGCTCTC**TGACGTCA**GCTCTC**TGACG**

The binding sites of NF-κb (underlined), CREB (bold), and HIF-1α (dotted) were labeled
