# Supplementary material for: Development of a computational promoter with highly efficient expression in tumors
Source: BMC Cancer. 2018 Apr 27;18:480. doi: 10.1186/s12885-018-4421-7 (PMC5924487; doi:10.1186/s12885-018-4421-7)
Supplement: Supplementary file 3 — The genes of regulate angiogenesis and cell growth. (PDF 102 kb) [file 12885_2018_4421_MOESM3_ESM.pdf]

Additional file 3. The genes of regulate angiogenesis and cell growth

**Angiogenesis & Cell growth**
GATA6 、LEF1 、NOV 、PKNOX1 、PML 、SOX17 、SRF 、XBP1
**Angioggenisis (GO:0001525)**
BRCA1 、E2F7 、CTNNB1 、E2F8 、EGR3 、ELK3 、EPAS1 、ETS1 、FOXC1 、FOXC2 、FOXO4 、GATA2 、GATA4 、GATA6 、GBX2 、GTF2I 、HAND1 、HAND2 、HDAC5 、HEY1 、HHEX 、HIF1A 、HIF3A 、HMGB1 、HOXA3 、HOXA5 、HOXA7 、HOXB13 、HOXB3 、ID1 、IL18 、ISL1 、JUN 、KDR 、KLF4 、KLF5 、LEF1 、MAP3K7 、MEIS1 、MEOX2 、NF1 、NFATC3 、NFATC4 、NOV 、NR2E1 、NR4A1 、OVOL2 、PITX2 、PKNOX1 、PML 、RBPJ 、SHH 、SOX17 、SOX18 、SPI1 、SRF 、STAT1 、TAL1 、TBX1 、TBX20 、TBX4 、TCF21 、TWIST1 、UBP1 、VEZF1 、XBP1 、ZNF304 、RORA
**Cell growth (GO:0016049)**
ABL1 、ADNP 、ADNP2 、AR 、BCL11A 、BCL6 、CREB1 、CREB3 、DNAJC2 、EMX1 、ENO1 、ESR2 、FOXK1 、FOXL2 、FOXM1 、FXN 、HNF4A 、IGFBP1 、ING1 、L1CAM 、LHX2 、LMX1A 、MSX1 、MYOCD 、NKX6-1 、NFKB1 、NOTCH2 、POU4F2 、POU4F3 、PPARD 、PPARG 、PRDM4 、RARG 、SIRT6 、SMAD3 、SMAD4 、SMARCA2 、SMARCA4 、SOX9 、TAF9 、TP53 、WT1 、ZNF639
