# Supplementary material for: Development of a computational promoter with highly efficient expression in tumors
Source: BMC Cancer. 2018 Apr 27;18:480. doi: 10.1186/s12885-018-4421-7 (PMC5924487; doi:10.1186/s12885-018-4421-7)
Supplement: Supplementary file 6 — Cooperativity analysis of NF-κB, CREB and HIF-1α. The protein-protein interaction of (A) NF-κB with CREB, (B) NF-κB with HIF-1α, and (C) CREB with HIF-1α were analyzed by GENEMANIA. The pink line indicates physical interaction, which is the highest interaction in the network. The purple, light blue, dark blue and green lines indicate that the protein has an interaction with the TF with different effects, co-expression, the involved pathway, co-localization and genetic interaction, respectively. (PDF 212 kb) [file 12885_2018_4421_MOESM6_ESM.pdf]

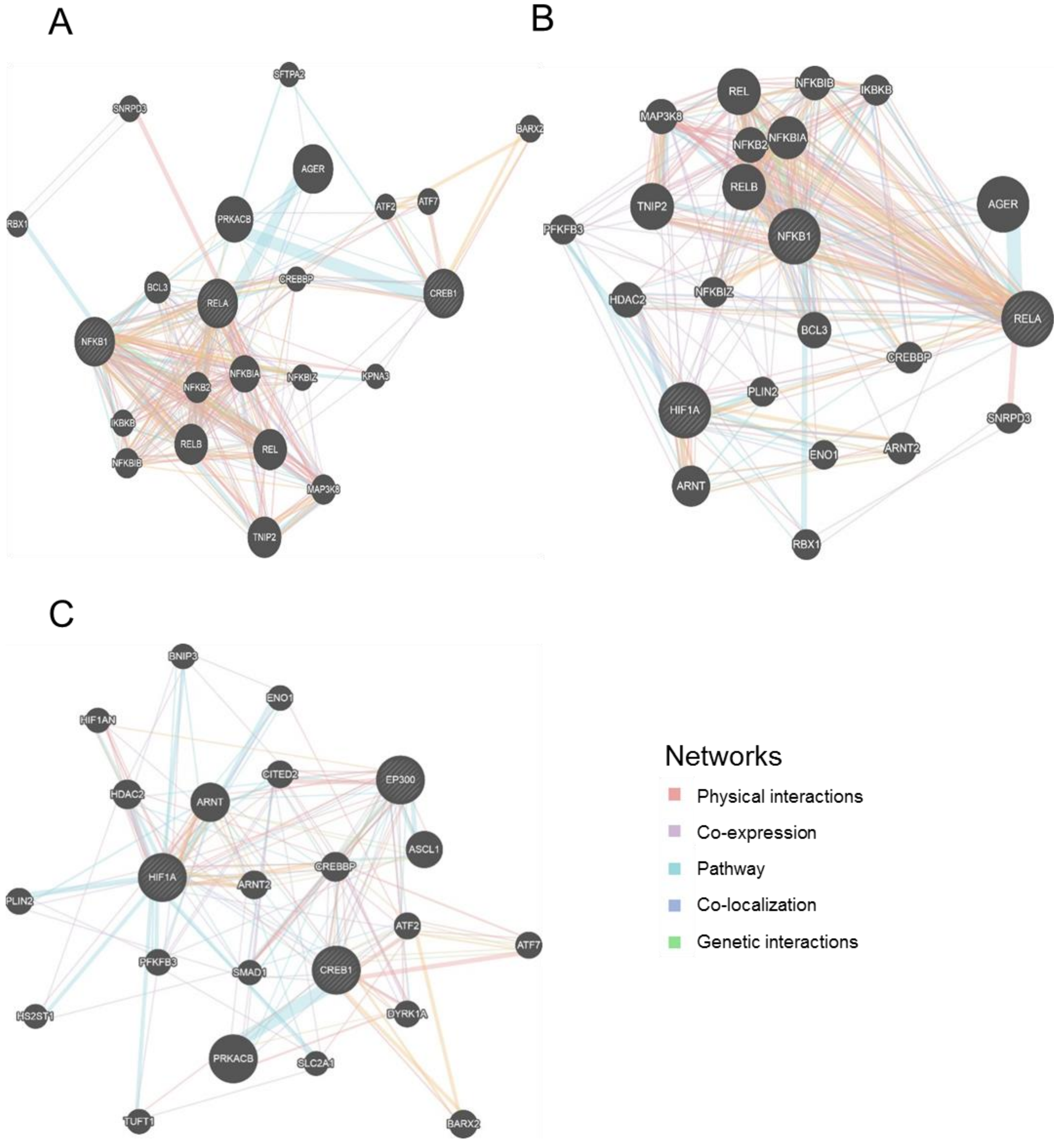
A
SFTPA2
SNRPD3
BARX2
AGER
ATF7
ATF2
PRKACB
RBX1
BCL3
CREBBP
CREB1
RELA
NFKB1
NFKBIA
NFKBIZ
KPNA3
NFKB2
IKBKB
RELB
REL
NFKBIB
MAP3K8
TNIP2
B
REL
NFKBIB
IKBKB
MAP3K8
NFKB2
NFKBIA
RELB
TNIP2
AGER
PFKFB3
NFKB1
HDAC2
NFKBIZ
BCL3
RELA
CREBBP
PLIN2
HIF1A
SNRPD3
ARNT2
ENO1
ARNT
RBX1
C
BNIP3
ENO1
HIF1AN
CITED2
EP300
HDAC2
ARNT
ASCL1
CREBBP
HIF1A
ARNT2
PLIN2
ATF2
ATF7
PFKFB3
SMAD1
CREB1
HS2ST1
DYRK1A
PRKACB
SLC2A1
TUFT1
BARX2
Networks
Physical interactions
Co-expression
Pathway
Co-localization
Genetic interactions

Additional file 6. Cooperativity analysis of NF-κB, CREB and HIF-1α**.** The protein-protein interaction of (A) NF-κB with CREB, (B) NF-κB with HIF-1α, and (C) CREB with HIF-1α were analyzed by GENEMANIA. The pink line indicates physical interaction, which is the highest interaction in the network. The purple, light blue, dark blue and green lines indicate that the protein has an interaction with the TF with different effects, co-expression, the involved pathway, co-localization and genetic interaction, respectively.
