# Supplementary material for: Development of a computational promoter with highly efficient expression in tumors
Source: BMC Cancer. 2018 Apr 27;18:480. doi: 10.1186/s12885-018-4421-7 (PMC5924487; doi:10.1186/s12885-018-4421-7)
Supplement: Supplementary file 7 — The expressive capabilities of D5 mini-promoter and CMV promoter in HEK293, B16F10 and HT29 cells. pD5-hrGFP or pCMV-hrGFP were transfected into (A) HEK293, (B) B16F10 or (C) HT29 cells 24 h, and the GFP expression intensities were detected by flow cytometer. The data were analyzed from three independent experiments, and the significant differences were calculated by t-test (* p < 0.05). (PDF 196 kb) [file 12885_2018_4421_MOESM7_ESM.pdf]

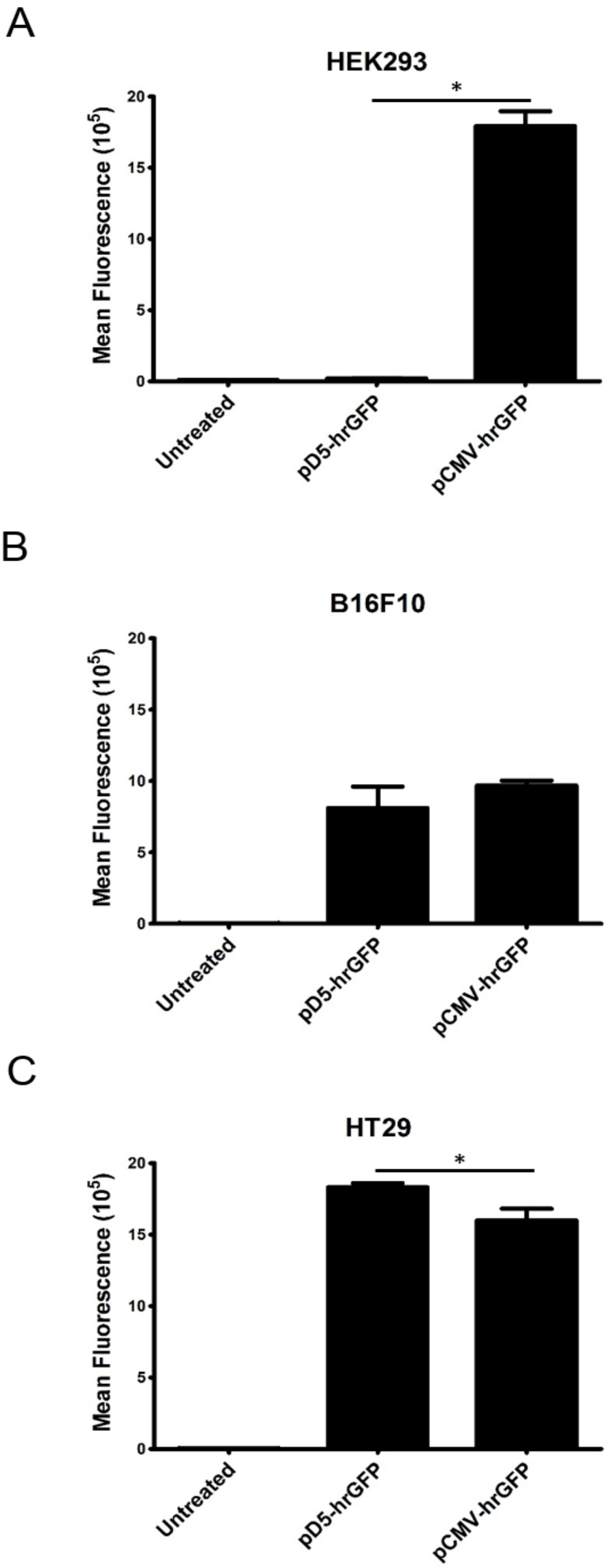
A
HEK293
*
Mean Fluorescence (10^5)
0
5
10
15
20
Untreated
pD5-hrGFP
pCMV-hrGFP
B
B16F10
Mean Fluorescence (10^5)
0
5
10
15
20
Untreated
pD5-hrGFP
pCMV-hrGFP
C
HT29
*
Mean Fluorescence (10^5)
0
5
10
15
20
Untreated
pD5-hrGFP
pCMV-hrGFP

Additional file 7. The expressive capabilities of D5 mini-promoter and CMV promoter in HEK293, B16F10 and HT29 cells. pD5-hrGFP or pCMV-hrGFP were transfected into (A) HEK293, (B) B16F10 or (C) HT29 cells 24 hours, and the GFP expression intensities were detected by flow cytometer. The data were analyzed from three independent experiments, and the significant differences were calculated by t-test (* $p<0.05$).
