# Supplementary material for: Development of a computational promoter with highly efficient expression in tumors
Source: BMC Cancer. 2018 Apr 27;18:480. doi: 10.1186/s12885-018-4421-7 (PMC5924487; doi:10.1186/s12885-018-4421-7)
Supplement: Supplementary file 8 — The effects of inhibitors for transcription factors on the hrGFP expression levels in PaTu8988T cells. pARE-hrGFP, pHIF-1α-hrGFP, pNFκB-hrGFP, pCRE-hrGFP and pD5-hrGFP were transfected into PaTu8988T cells, respectively. The pD5-hrGFP-transfected cells were treated respectively with different inhibitors, 30 μM Bay11-7082 (NFκB inhibitor), 1 μg/ml DMGF (CREB inhibitor) and 1 atm oxygen. Twenty-four hours after transfection, the green fluorescent levels of hrGFP were determined by flow cytometer. The data were calculated and analyzed from three independent experiments, and the significant differences were calculated by t-test for the pD5-hrGFP transfected cells v.s. the pD5-hrGFP transfected cells treated with inhibitor (*p < 0.05). (PDF 233 kb) [file 12885_2018_4421_MOESM8_ESM.pdf]

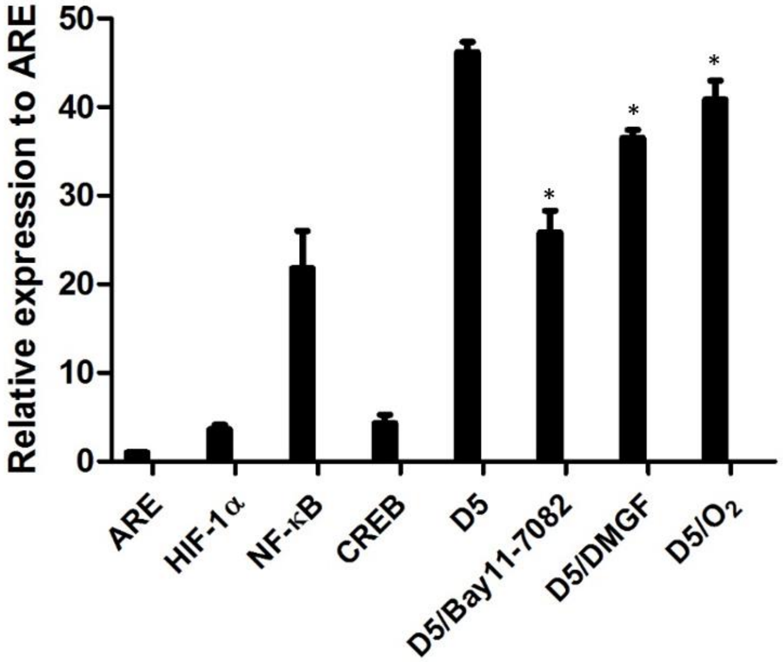

Additional file 8. The effects of inhibitors for transcription factors on the hrGFP expression levels in PaTu8988T cells. pARE-hrGFP, pHIF-1α-hrGFP, pNFκB-hrGFP, pCRE-hrGFP and pD5-hrGFP were transfected into PaTu8988T cells, respectively. The pD5-hrGFP-transfected cells were treated respectively with different inhibitors, 30μM Bay11-7082 (NFκB inhibitor), 1μg/ml DMGF (CREB inhibitor) and 1 atm oxygen. Twenty-four hours after transfection, the green fluorescent levels of hrGFP were determined by flow cytometer. The data were calculated and analyzed from three independent experiments, and the significant differences were calculated by t-test for the pD5-hrGFP transfected cells v.s. the pD5-hrGFP transfected cells treated with inhibitor (*$p<0.05$).
