# Supplementary material for: Development of a computational promoter with highly efficient expression in tumors
Source: BMC Cancer. 2018 Apr 27;18:480. doi: 10.1186/s12885-018-4421-7 (PMC5924487; doi:10.1186/s12885-018-4421-7)
Supplement: Supplementary file 9 — Histopathologic analysis of mice treated with LPPC/DNA complexes. (A) H&E staining of normal organs including the heart, liver, spleen, lung, kidney, intestine and stomach at day 7 after an intravenous injection of PBS, LPPC/pCMV-RDBV-IgG1 Fc, LPPC/ pCMV-IgG1 Fc, pD5-RDBV-IgG1 Fc or LPPC/pD5-IgG1 Fc complexes. (Scale bar: 50 μm, 400×) (B) IHC staining of normal organs using anti-Human IgG1 Fc antibody. (Scale bar: 50 μm, 400×) (C) Statistical analysis of the average score of human IgG1 Fc staining normal organs. Significance differences were evaluated by t-test, and the p values were represented as the tested group compared with the control group (*: p < 0.05). (PDF 1022 kb) [file 12885_2018_4421_MOESM9_ESM.pdf]

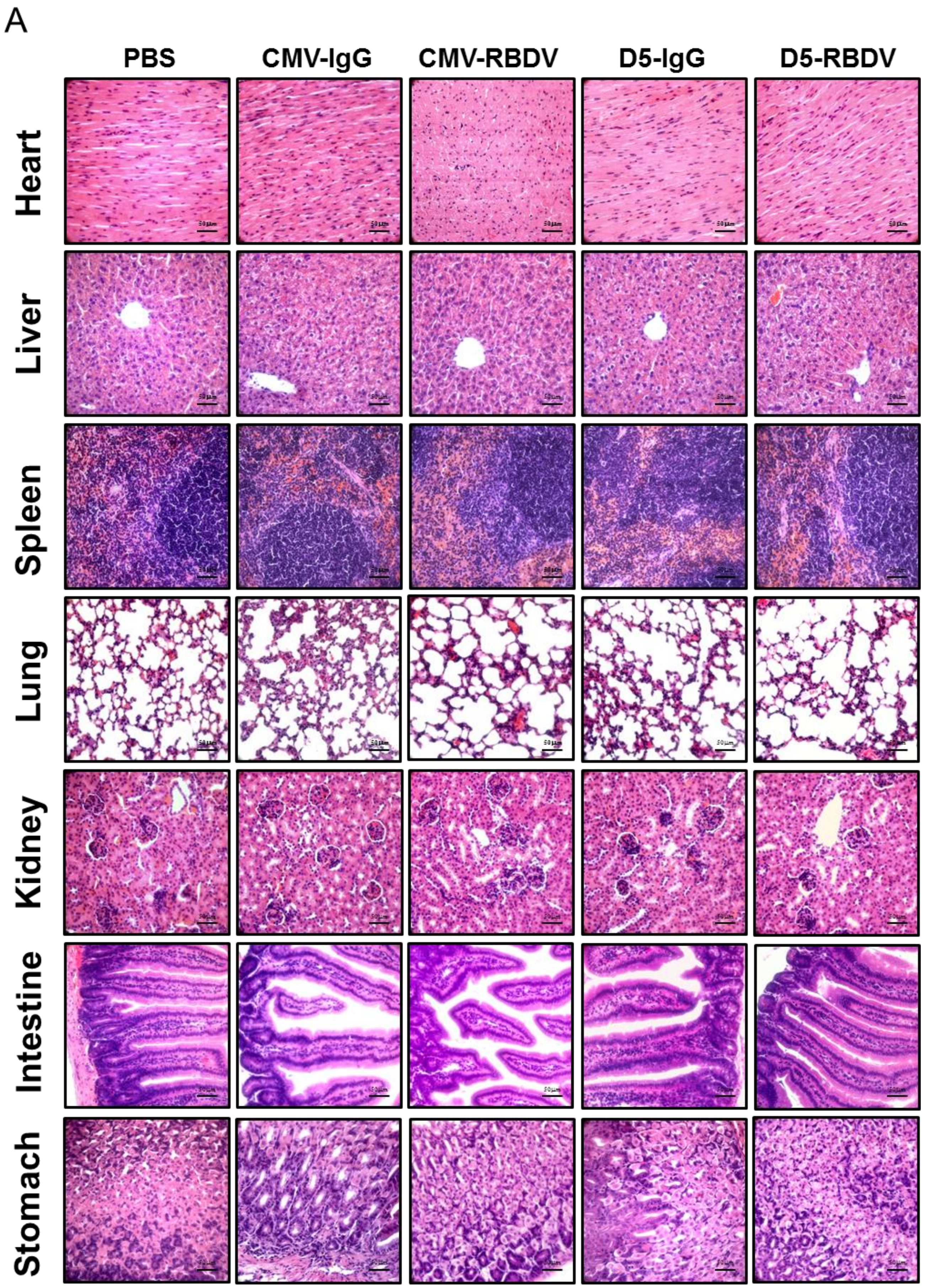
A
PBS
CMV-IgG
CMV-RBDV
D5-IgG
D5-RBDV
Heart
Liver
Spleen
Lung
Kidney
Intestine
Stomach

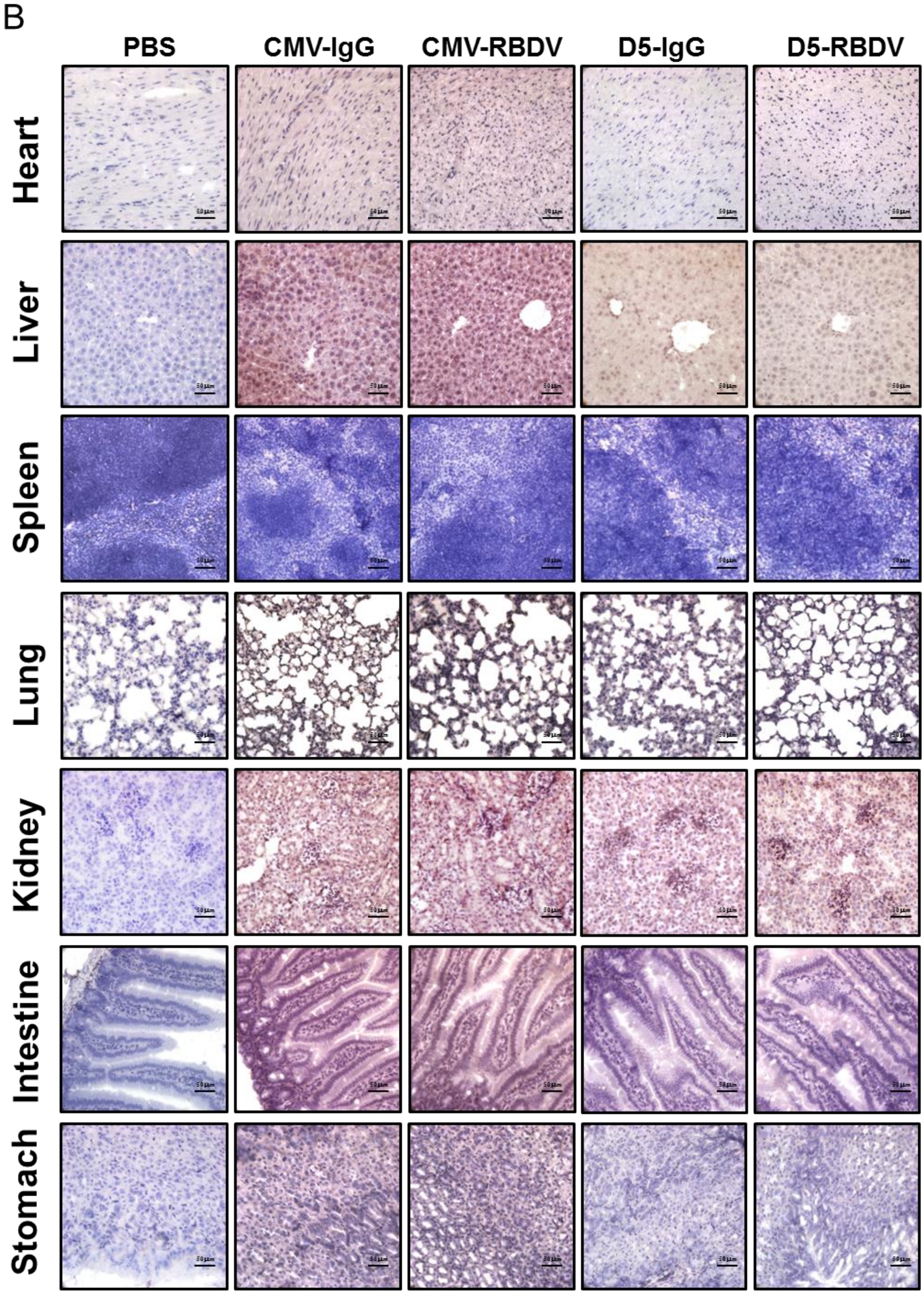
B
PBS
CMV-IgG
CMV-RBDV
D5-IgG
D5-RBDV
Heart
Liver
Spleen
Lung
Kidney
Intestine
Stomach

C

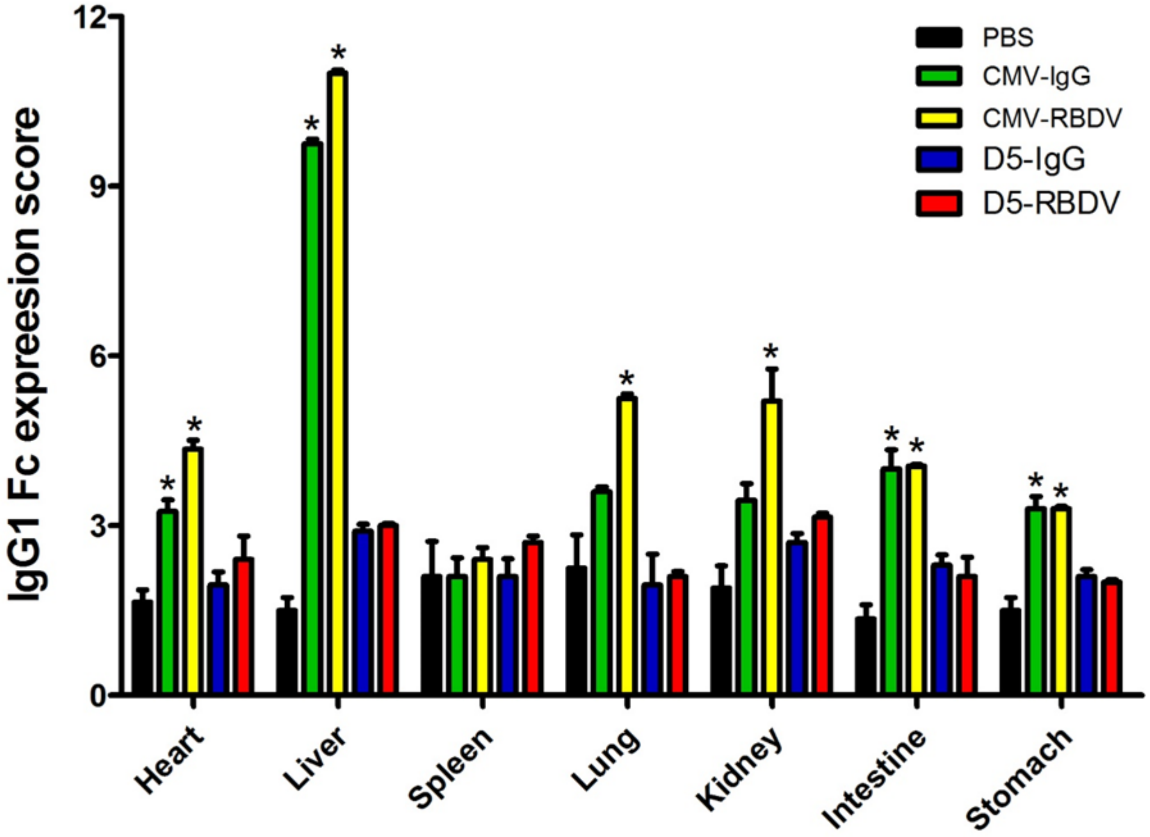

Additional file 9. Histopathologic analysis of mice treated with LPPC/DNA complexes. (A) H&E staining of normal organs including the heart, liver, spleen, lung, kidney, intestine and stomach at day 7 after an intravenous injection of PBS, LPPC/pCMV-RDBV-IgG1 Fc, LPPC/ pCMV-IgG1 Fc, pD5-RDBV-IgG1 Fc or LPPC/ pD5-IgG1 Fc complexes. (Scale bar: 50 μm, 400×) (B) IHC staining of normal organs using anti-Human IgG1 Fc antibody. (Scale bar: 50 μm, 400×) (C) Statistical analysis of the average score of human IgG1 Fc staining normal organs. Significance differences were evaluated by t-test, and the p values were represented as the tested group compared with the control group (*: $p<0.05$).
